# Antibiotics and mental health: The good, the bad and the ugly

**DOI:** 10.1111/joim.13543

**Published:** 2022-07-12

**Authors:** Katherine Dinan, Timothy Dinan

**Affiliations:** ^1^ School of Medicine Trinity College Dublin Ireland; ^2^ Department of Psychiatry and APC Microbiome Ireland University College Cork Cork Ireland

**Keywords:** antibiotics, brain–gut axis, depression, gut microbiota, microbial diversity, microglia

## Abstract

Antibiotics are recognised as, on occasion, producing psychiatric side effects, most notably depression and anxiety. Apart from antimicrobial activity, antibiotics have multiple off‐target effects. The brain–gut–microbiota axis has multiple sites for off‐target activity, which may produce either positive or negative antibiotic effects. Here we review how antibiotics impact mental health by acting through the brain–gut–microbiota axis. Microbes in the gut influence brain function by acting through the vagus nerve or by altering the production of short‐chain fatty acids or the amino acid tryptophan, the building block of serotonin. Not all antimicrobial actions of antibiotics have a negative impact. The first antidepressant discovered was actually an antibiotic: isoniazid is an antibacterial drug developed for treating tuberculosis. Minocycline, which enters the brain and mediates its effects through microglia, shows antidepressant activity. Some antibiotics bring about a significant decrease in gut microbial diversity, and this is viewed as a risk factor for depression. Other risk factors induced by antibiotics include altered gut barrier function, activation of the hypothalamic–pituitary–adrenal axis, reducing levels of brain‐derived neurotrophic factor or oxytocin and alteration of vagal tone. Although most patients taking antibiotics do not suffer from an iatrogenic psychiatric disorder, some do. As clinicians, we need to keep this in mind. The development of new antibiotics is primarily focused on antibiotic resistance, but efforts should be made to reduce off‐target brain–gut–microbiota effects resulting in mental health problems.

## Graphical abstract

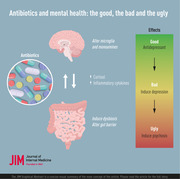

The view that antibiotics, bacteria and mental health are related may seem fanciful at face value. There is no doubt that from a health perspective, antibiotics were the single most important pharmacological discovery of the 20th century. However, apart from targeting disease‐causing bacteria, most antibiotics have multiple off‐target effects both on non‐disease‐related bacteria and host physiology. Given the fact that the gut contains the greatest concentration of bacteria in the body, it is not surprising that it is frequently a target for antibiotic activity. Broad‐spectrum antibiotics prescribed for 1 week can alter gut microbes for several weeks [1]. The concept of the holobiont has been developed to describe the ecological unit comprising both the host species and its symbiotic microbiota [2]. Antibiotics impact the holobiont both at a microbial and host cellular level, and the impact on mental health is the product of such an interaction.

The adult intestine is estimated to contain over 1 kg of bacteria, approximately the same weight as the human brain, and this is called the gut microbiota [3]. It is a core part of what is now referred to as the brain–gut–microbiota axis [4], considered to be responsible for many of the positive and negative mental health aspects of antibiotics [5, 6, 7]. This review will discuss the ways in which antibiotics impact mental health by acting through the brain–gut–microbiota axis.

## Architecture of gut microbiota

Until recently, it was estimated that we had 10 times more bacteria than human cells in our bodies. More accurate calculations put the relative ratio at slightly over 1 microbe to every human cell [8]. Over a thousand strains of bacteria have been identified in the gut microbiota [9, 10, 11]. Many of these strains remain unculturable and have been identified by sequencing. However, at a genomic level, the overwhelming majority of genes in our bodies are microbial, calculated to be in excess of 10 million microbial genes [12, 13, 14]. The fine architecture of the healthy microbiota is unique to each individual. Intra‐individual differences over time are far less significant than the differences between individuals [15, 16]. In a healthy individual, the maintenance of homeostasis is essential for a balanced compositional signature [17, 18, 19]. When this homeostasis is, for whatever reason, disrupted, disease vulnerability emerges from many different sources [20].

Despite the complexity of the individual gut microbiota, some researchers maintain that it is possible to classify the human gut microbiota into different enterotypes [21, 22, 23]. Such attempts are contentious and criticised as being an oversimplification [24]. Three discrete enterotypes have been defined, each on the basis of a high level of a single microbial genus. The enterotypes are *Bacteroides spp., Prevotella spp*. or *Ruminococcus spp*. [21, 25]. It is claimed that these enterotypes are functionally distinct. For example, the *Bacteroides spp*. enterotype is characterised by high fat or protein diets, and the *Prevotella spp*. enterotype with high‐carbohydrate diets [26].

Overall, the main determinant of gut microbiota architecture in adulthood is diet and, to a lesser extent, exercise but medications, especially antibiotics, play an important role [27].

## How do gut microbes communicate with the brain?

**Fig. 1:**
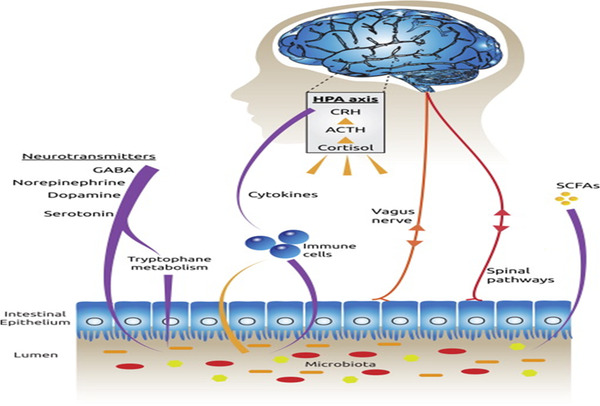
Connections between the gut and brain, including the vagus nerve, SCFAs such as butyrate, cytokines and tryptophan, which is the building block for serotonin (from Cryzan and Dinan [114]). Abbreviations: ACTH, adrenocorticotropic hormone; CRH, corticotrophin‐releasing hormone; GABA, gamma aminobutyric acid; HPA, hypothalamic–pituitary–adrenal; SCFAs, short‐chain fatty acids.

There are a number of parallel routes through which gut microbes communicate with the brain [28, 29]. The vagus nerve, that long meandering nerve linking the brain with other internal organs, is a key channel for information transfer (Fig. 1). It sends signals from the brain to the gut and from microbes to the brain. Bravo et al. showed that in rodents, *Lactobacillus rhamnosus* (JB1 strain) impacted behaviour by acting via gamma aminobutyric acid (GABA) receptors in multiple brain regions [30]. However, when animals underwent a full truncal vagotomy, no such effects were observed. It seems reasonable to conclude that certain microbes only communicate with the brain when the vagus nerve is intact. Interestingly, those who previously underwent vagotomy for treatment of peptic ulcer disease may be less likely to develop Parkinson's disease [31], providing human data support for the importance of the vagus nerve in linking the gut and brain.

Short‐chain fatty acids (SCFAs) such as butyrate and propionate are the products of bacterial metabolic activity [32, 33]. Humans cannot produce these in adequate quantities, but nonetheless, they are essential for maintaining normal metabolic activity. Structurally, they are carboxylic acids with aliphatic tails of 1–6 carbon [34]. They are produced by the bacterial fermentation of complex polysaccharides, which we are unable to digest. The overwhelming majority of SCFAs produced in the gut are acetate, propionate and butyrate, but small amounts of valerate, isovalerate, valproate, caproate, isocaproate, succinate, isobutyrate and hexanoate are also synthesised [35]. They act through G‐protein coupled receptors (GPCRs). There are two key GPCRs mediating such activity—free fatty acid receptor 2 (FFAR2) and FFAR3 [36]. However, they also act as epigenetic modulators, inhibiting histone deacetylase (HDAC) [37], and this may be more important in terms of brain activity than the action through GPCRs [38]. It has been speculated that they reach the brain via the bloodstream, but this has yet to be definitively shown using nonpharmacological doses. SCFAs are metabolised in the liver, so much smaller quantities reach the brain.

Tryptophan from the diet has long been known to play a crucial role in brain serotoninergic activity. The human brain has limited storage capacity for this amino acid. Tryptophan is the building block for serotonin [39]. However, it has now been shown that bifidobacteria within the intestine can synthesise tryptophan and that administration of certain bifidobacteria is associated with increased plasma levels of the amino acid [40]. Tryptophan, either produced by gut microbes or derived from the diet, is metabolised by indoleamine 2,3‐dioxygenase 1 (IDO1) that is present in the gut mucosa and leads to increased blood kynurenine levels, which can cross the blood–brain barrier. Certain microbes, such as *Lactobacillus reuteri*, have the ability to downregulate IDO1 in the gut mucosa and thus influence levels of circulating kynurenine [41].

As well as tryptophan, gut microbes produce all of the key central neurotransmitters such as GABA, norepinephrine, dopamine, glutamate and histamine [42, 43]. GABA, for instance, is produced by all lactobacilli, some producing more than others. While these neurotransmitters do not reach the brain, and even if they did cannot cross the blood–brain barrier, they can influence the brain by signalling through the enteric nervous system and perhaps signalling through the vagus nerve. Antibiotics that negatively impact lactobacilli consequently decrease GABA production and activity in the gut [44].

Studies in rodents have demonstrated the capacity of health‐promoting bacteria such as bifidobacteria in damping activity in the core stress system, namely, the hypothalamic–pituitary–adrenal (HPA) activity. In contrast, cytokines such as IL‐1 and IL‐6 produced by immune cells in the gut mucosa in response to certain strains of pathogenic gut bacteria can activate the HPA axis [45]. Of note, increased activation of the HPA in turn, increases circulating levels of kynurenine by activation of the hepatic enzyme, tryptophan 2,3 dioxygenase (TDO2).

## Antibiotics as antidepressants

It is often forgotten that the first antidepressant discovered was actually an antibiotic. Isoniazid is an antibacterial drug developed in the United States in the 1950s for treating tuberculosis [46]. Unexpected side effects of euphoria, psychostimulation, increased appetite and improved sleep prompted an interest in the medication as a potential treatment for depression. A physician treating patients with tuberculosis originally observed these findings [46]. Subsequent clinical trials confirmed antidepressant efficacy, which was attributed to isoniazid's ability to inhibit monoamine oxidase (MAO) enzymes and, therefore, increase levels of monoamines such as noradrenaline, serotonin and dopamine in the brain. This observation gave rise to the ‘monoamine hypothesis of depression’, which is still widely researched and resulted in the development of several classes of antidepressant medications, including tricyclic antidepressants (TCAs) and serotonin‐specific reuptake inhibitors (SSRIs), both of which act to increase central monoamine levels. Recent studies indicate that newer antidepressants, for example, the SSRI, fluoxetine, have antibiotic activity in certain circumstances [47].

Kohler‐Forsberg et al. [48] conducted a meta‐analysis of clinical trials of anti‐inflammatory drugs, including the antibiotic minocycline, in depressed patients. They concluded that minocycline as an adjunctive therapy positively impacted depressive symptoms. In a subgroup of patients who had psychotic depression, Miyaoka et al. also found efficacy when the antibiotic was administered as an adjunctive therapy [49]. More recently, Zazula et al. [50] pooled the data from two studies and reached similar conclusions. However, Husain et al. found no evidence of minocycline efficacy in bipolar depression [51]. The data, therefore, support a role for minocycline in the adjunctive treatment of unipolar depression but fails to find efficacy in bipolar patients.

In summary, some antibiotics, such as minocycline, are reported as having antidepressant actions, and some antidepressants are antibiotic.

## Antibiotics inducing psychiatric illness

That antibiotics can negatively impact mental health is based on a variety of observations both preclinically and clinically. In a preclinical study, Lach et al. examined the behavioural effect of gut microbiome depletion with antibiotics [52]. They explored the impact of 3‐week microbiota depletion with antibiotics during the adolescent period and in adulthood. Following a washout period to restore the gut microbiota, behavioural and molecular markers of gut–brain communication were investigated. The data showed that transient microbiota depletion in adolescence had long‐lasting effects on microbiota architecture and importantly increased anxiety‐like behaviour in mice exposed to antibiotic treatment during adolescence but not when exposed in adulthood. This is a very important observation. Similarly, gene expression in the amygdala, a brain region associated with anxiety, was more severely affected in mice treated during adolescence. The study demonstrates the vulnerability of the gut microbiota during this critical adolescent period and the long‐lasting impact manipulations of the microbiota can have on gene expression and behaviour into adulthood. There is now unequivocal evidence that babies exposed to antibiotics in the first year of life are far more likely to grow up as obese adults and in childhood, are more likely to suffer from asthma, allergies and attention deficit hyperactivity disorder [53]. Both obesity and attention deficit hyperactivity disorder are thought to be mediated by the brain–gut–microbiota axis.

The first report of antibiotic‐induced depression was published as recently as 2010 [54]. A 75‐year‐old man without a history of psychiatric illness became acutely depressed and subsequently committed suicide. He had received antibiotic treatment for a postoperative wound infection following colorectal cancer surgery. The treatment consisted of levofloxacin and trimethoprim sulphamethoxazole and this was considered the principal cause of his suicide at the coroner's court.

Based on a series of nested case‐control studies carried out between 1995 and 2013 using a large population‐based medical record database from the UK, Lurie et al. concluded that single antibiotic treatment regimens, such as penicillin and quinolones, can cause an increase in the risk of depression and anxiety [55]. The study focused on a broad range of mental health outcomes, not just depression and anxiety. The study included 202,974 patients with depression, 14,570 with anxiety, 2690 with psychosis and 872,462 matched controls. Cases were defined as individuals aged 15–65 years with any medical diagnosis of depression, anxiety or psychosis. Patients taking psychotropic drugs within 90 days of the index date were excluded. For every case, four controls were selected, matching for age, sex, practice site, time of the year and duration of follow‐up. The primary outcome variable was therapy with one of seven antibiotic classes more than 1 year before the index date. Odds ratios and 95% confidence intervals (CIs) were calculated for the association between each psychiatric disorder and prescription of an antibiotic. Data were analysed using a conditional logistic regression model. The following variables were included as covariates: obesity, smoking history, alcohol consumption, socioeconomic status and the number of infections before diagnosis.

They found that treatment with a single antibiotic course was associated with a higher risk for depression in all antibiotic groups, with an adjusted odds ratio of 1.23 for penicillins (95% CI, 1.18–1.29) and 1.25 (95% CI, 1.15–1.35) for quinolones. The risk increased with repeat antibiotic exposure to 1.40 (95% CI, 1.35–1.46) and 1.56 (95% CI, 1.46–1.65). A similar relationship was established for anxiety and was most prominent with exposures to penicillins and sulfonamides, with an odds ratio of 1.17 (95% CI, 1.01–1.36) for a single course of penicillin and 1.44 (95% CI, 1.18–1.75) for more than five courses. Interestingly, they found no change in the risk for psychosis with any antibiotic. There was a slight increase in the risk of depression and anxiety with a single course of antifungals; however, there was no increase in risk with repeated exposures. The overall conclusion was that antibiotic exposure increases the risk for depression and anxiety but not for psychosis.

Recently, similar results have been reported in a clinical study based on fluoroquinolones. In a survey, 94 patients who took fluoroquinolones reported the following psychiatric side effects: anxiety disorder (72%), depression (62%), insomnia (48%), panic attacks (37%) and cognitive impairment (33%) [56]. In contrast, Klein‐Petersen et al. also report a number of cases of psychosis associated with antibiotic administration [57]. They found an association between the fluoroquinolones and neurotoxic adverse events with a schizophrenia‐like syndrome. They advocated for large‐scale longitudinal studies to explore the issue in greater detail.

Thus, it would seem that antibiotics increase the risk of depression and anxiety, while the risk of psychosis is far less and associated with a limited class of antibiotics. The risk is greater when multiple antibiotics are prescribed or where repetitive prescriptions are required. However, it is clear that most patients who are prescribed these antibiotics do not develop psychiatric complications. It has not been established why some patients may be at a greater risk.

Here we explore the impact of antibiotics on the brain–gut–microbiota axis and the negative consequences on mental health.

## Off‐target brain–gut–microbiota actions

### Direct brain action

Minocycline hydrochloride is a broad spectrum second‐generation derivative of the bacteriostatic antibiotic tetracycline. Both share a common core of four six‐membered rings. Minocycline has modifications at three sites on the conserved structure compared to tetracycline. These modifications to the core structure increase the half‐life in comparison to first‐generation antibiotics and improved absorption. The structural alterations also enable penetration of the blood–brain barrier.

Having penetrated the blood–brain barrier, minocycline shows considerable activity, especially in impacting microglia [58]. Two polar opposite phenotypes of microglia have been described: pro‐inflammatory (M1) and anti‐inflammatory (M2). In reality, microglia, when in homeostasis, probably exist on a continuum between these two extremes. Minocycline, when it crosses the blood–brain barrier, has been shown to inhibit microglial activation. This is an off‐target effect that can be used for therapeutic purposes. As discussed earlier, minocycline may be of use in treating depression, but it has also been tested in a variety of neurological disorders, including amyotrophic lateral sclerosis [59].

Clarithromycin, beta‐lactams and fluoroquinolones block GABA‐A transmission, and because of this off‐target effect clarithromycin has been used to treat hypersomnolence [60, 61]. Linezolid, the oxazolidinone, impacts CNS activity by inhibiting MAO [62], as do metronidazole and isoniazid. These antibiotics should be prescribed with caution in those taking selective serotonin reuptake inhibitors or TCAs. Glutamate is the key excitatory neurotransmitter, and NMDA receptors within this system have been found to be a target for some antibiotics [63, 64]. D‐cycloserine and aminoglycosides are recognised as binding to NMDA sites. There is some evidence of efficacy in treating depression and anxiety [65], but it fails to show efficacy in difficult‐to‐treat schizophrenia [66]. There are early preclinical data to indicate potential in autism [67].

### Decreased microbial diversity

Increasing evidence supports the view that depression is associated with decreased microbial diversity and that transplant of such a microbiota into rodents results in depressive‐like behaviours and alterations in tryptophan metabolism [68, 69]. To understand acute and chronic antibiotic‐induced changes in the gut microbiota of healthy adults, Anthony et al. [70] sequenced the microbiome before, during and 6 months after exposure to four common antibiotic regimens. They found an acute decrease in species richness and culturable bacteria after antibiotics. In almost all cases, a healthy adult microbiome returned to pretreatment architecture after 2 months. However, they observed an altered taxonomy, resistome and metabolism with an increased antibiotic resistance level.

Azithromycin, the azalide (a subclass of macrolide), causes a delayed recovery of species richness. This antibiotic is associated with an increased risk of depression [71]. In the latter study, a subset of subjects showed a persistent reduction in microbial diversity after antibiotics and had compositional similarities to patients with a diagnosis of major depression. These findings increase our understanding of how antibiotics impact microbiome dynamics, resilience and recovery and provide strong support for the view that changes in gut microbial diversity are central in the pathophysiology of depression induced by antibiotics.

### Alteration of the HPA axis

There is increasing evidence to support the view that the gut microbiota plays a role in regulating the body's major neuroendocrine stress system, the HPA axis [72]. This is the core stress axis in humans and is implicated in a variety of psychiatric disorders. It is well established that overactivation of the HPA is associated with severe forms of depression. Antidepressants reduce HPA activity. The interleukin cytokines IL‐1 and IL‐6 are the most potent activators of the HPA [73]. Given the fact that the gut microbiota and HPA are in bidirectional communication, it is hardly surprising that antibiotics acting on the gut microbiota influence HPA activity. Antibiotics in certain circumstances may activate the HPA and induce stress‐related disorders. Such a view is supported by data from germ‐free animals, who lack a microbiota [74]. These animals have increased corticosterone production. As far back as 1952, a case report described stimulation of the HPA in response to penicillin [75]. If this likely mechanism is correct, it leads to the conclusion that antibiotic exposure in a setting of major stress, either psychological or physical, would significantly increase the risk of depression. This hypothesis has not been directly tested. There is, however, evidence to support the view that the antibiotic minocycline damps down HPA activity, which may partly explain its antidepressant action [76].

### Production of neurotransmitters

All of the major neurotransmitters can be produced by gut microbes [77]. For example, lactobacilli all produce GABA to varying degrees [62, 78]. It is also well established that gut microbes influence the serotonergic system [79]. Germ‐free animals and those treated with broad‐spectrum antibiotics show significant alterations in central serotonergic activity. Bifidobacteria are a group of lactic acid bacteria that includes the putative probiotic strains of the *Lactobacillus*, *Bifidobacterium* and *Enterococcus* genera [80]. Bifidobacteria have been shown to increase blood tryptophan levels, which in turn regulates 5HT synthesis [40]. This system plays a fundamental role in regulating mood, sleep and appetite [81]. All strains of bifidobacteria, whatever the species, are relatively sensitive to penicillins, including penicillin G, amoxicillin, piperacillin, ticarcillin, imipenem and also antibiotics such as macrolides, clindamycin, pristinamycin, vancomycin and teicoplanin [82]. Given the fact that bifidobacteria are regarded as having a mental health benefit and strains of bifidobacteria have been shown to impact stress responses [83, 84], it is tempting to speculate that this action of antibiotics may have negative mental health consequences. In support of this view is the fact that in a rodent study, both clindamycin and amoxicillin were found to increase depressive‐like behaviours [85].

### Modulation of brain‐derived neurotrophic factor

The myokine brain‐derived neurotrophic factor (BDNF) is known to be altered in depression and levels are significantly altered in germ‐free animals who have no microbiota [86, 87, 88]. Most studies find that BDNF levels are low in depressed patients and are increased by antidepressants such as fluoxetine [89]. Bistoletti et al. [90] evaluated the expression of BDNF and its high‐affinity receptor tropomyosin‐related kinase B (TrkB). The study was conducted in juvenile mice following 2 weeks of antibiotic treatment. A cocktail of broad‐spectrum antibiotics was used consisting of vancomycin 50 mg/kg, neomycin 10 mg/kg, metronidazole 100 mg/kg and ampicillin 10 mg/kg. In both, the mucosa and mucosa‐deprived whole‐wall small intestine segments of the antibiotic‐treated animals, BDNF and TrKB mRNA and protein levels significantly increased. In the longitudinal muscle‐myenteric plexus preparations, the percentage of myenteric neurons staining for BDNF and TrkB was increased. BDNF and TrkB protein levels were lower in the hippocampus than in control animals. Immunostaining for BDNF and TrkB showed similar alterations in the hippocampal pyramidal CA3 and dentate gyrus subregions. The data indicate that depleting the gut microbiota differentially influenced the expression of BDNF‐TrkB in the juvenile mice in both the enteric and central nervous systems. The study has obvious implications for the use of broad‐spectrum antibiotics in neonates.

Ceftriaxone, the third‐generation cephalosporin, is known to be a glutamate transporter subtype 1 (GLT‐1) enhancer [91]. In a rodent study, it was found to significantly impact the gut microbiota and reduce BDNF levels in the hippocampus [92]. These are changes viewed as consistent with those of major depression, and ceftriaxone is known to cause depression.

### Oxytocin

Oxytocin is a peptide released from the posterior pituitary and is also active in limbic regions of the brain [93, 94]. It has been implicated both in depression, autism and social anxiety disorders [95, 96, 97]. In patients with major depression, Scantamburlo et al. [98] found that plasma oxytocin levels were reduced compared to individuals without a mood disorder and oxytocin has been reported as possessing prosocial effects [99]. There is increasing evidence from rodent studies that gut microbes can alter oxytocin levels, and early exposure to antibiotics may increase the risk of autism [100, 101, 102]. In a study in mice, Desbonnet et al. found that early exposure to broad‐spectrum antibiotics leads to a decrease in brain oxytocin levels [103]. It has been speculated that the impact of antibiotics on mental health may be mediated through the oxytocin system. However, we could not find any published data from human studies to directly support this hypothesis.

### Adjustment of vagal tone

Vagal tone may play a role in depression. In support of this is the fact that in severe cases, depression is treated by vagal nerve stimulation [104]. Certain microbes also use the vagus nerve to communicate with the brain. This is true of *L. rhamnosus* (JB1), which may have antidepressant actions [30]. The extent to which antibiotics influence vagal tone has not been directly explored. The vagal nerve, when stimulated, does produce anti‐inflammatory activity, which in turn may have an antidepressant effect. Wang et al. did show in a rodent model that a subdiaphragmatic vagotomy ameliorated the effects of antibiotic‐induced behavioural changes [105]. No direct human data are currently available to support the view that antibiotics may alter the vagal tone.

### Production of SCFAs

SCFAs are not synthesised by humans and are only derived from the gut microbiota metabolism. Some SCFAs, especially butyrate, are an important energy source for intestinal epithelium cells, and butyrate plays a major role in the maintenance of hypoxia at the epithelial border of the intestine. Short SCFAs have significant epigenetic effects, as described above. Butyrate, in particular, has been shown to be an HDAC inhibitor, but also acts through GPCRs, namely FFAR2 and FFAR3. A wide variety of broad‐spectrum antibiotics have a profound impact on butyrate levels [106]. Several groups have shown that a course of treatment with broad‐spectrum antibiotics can affect the abundance and colonisation of intestinal flora and bring about a decrease in the content of SCFAs. This is especially the case in low birth weight infants and, as a result, may have developmental consequences [107]. Romick‐Rosendale et al. [108] carried out a major longitudinal examination of changes in the gut SCFAs in children undergoing allogeneic hematopoietic stem cell transplantation and the relationship of those changes to the microbiota and antibiotic exposure. They found significant alterations in butyrate and other SCFA levels, which were lower in children receiving antibiotics with activity against anaerobic organisms. While in healthy adults, the administration of either clindamycin or ampicillin resulted in a significant decrease in SCFA levels [109].

**Fig. 2:**
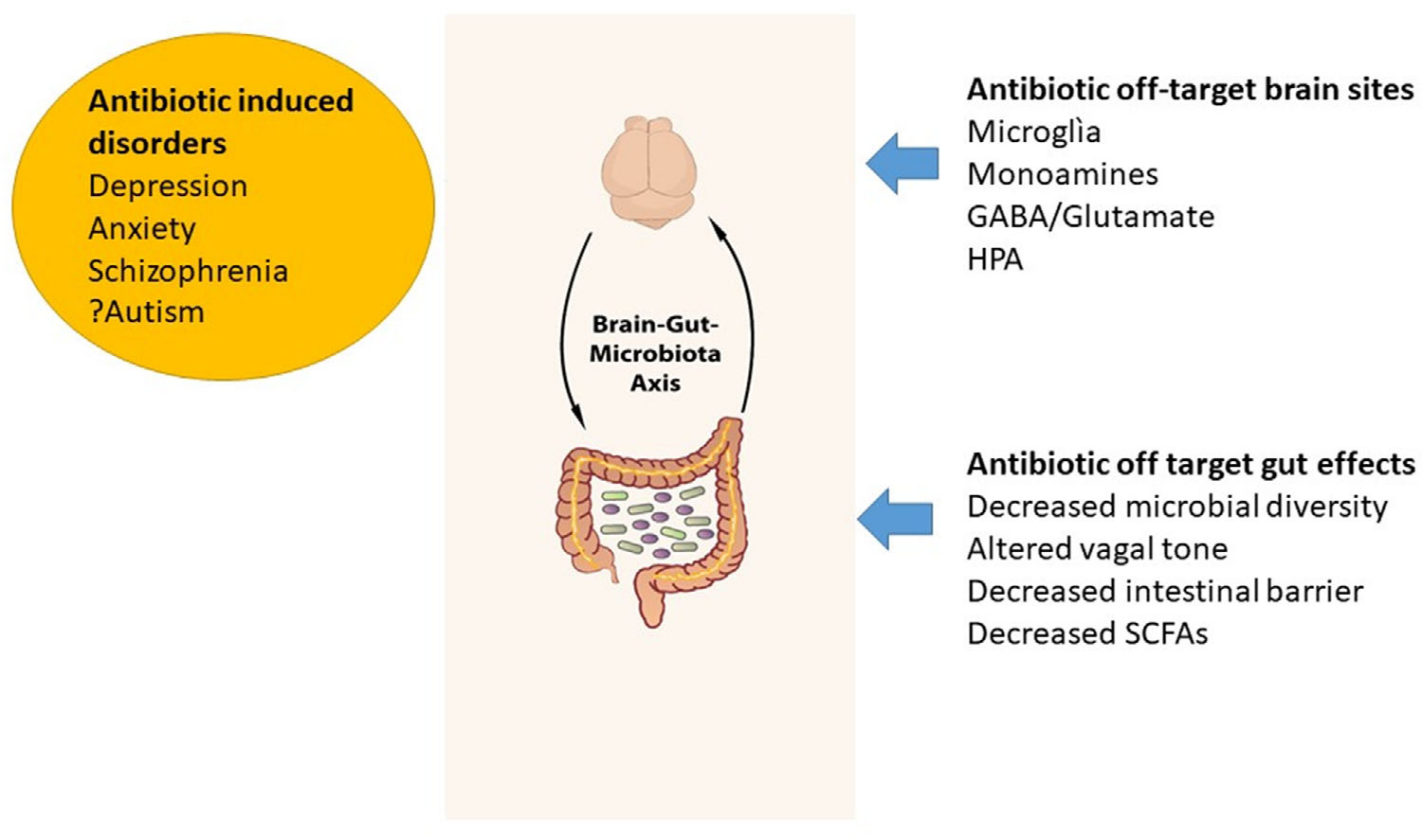
The antibiotic off‐target sites in both the brain and gut, together with the psychiatric disorders associated with antibiotics. Abbreviations: GABA, gamma aminobutyric acid; HPA, hypothalamic–pituitary–adrenal; SCFAs, short‐chain fatty acids.

Overall, the data provide evidence that antibiotic use can induce dysbiosis and alter essential SCFA levels (Fig. 2).

### Continuity of the intestinal barrier

Tight junction barrier function is critical to intestinal homeostasis and is maintained by a variety of proteins, including occludins. Feng et al. [110] investigated the effects of antibiotics on the intestinal tight junction barrier and the related mechanisms. Healthy adult female mice were treated with a cocktail of broad‐spectrum antibiotics (ampicillin, neomycin, metronidazone and vancomycin) for 14 days. The microbiota was assessed using 16S rDNA sequencing and gas chromatography, and mass spectrometry was used to determine SCFA levels. Protein expression was measured by immunoblotting. Antibiotics induced a gut dysbiosis in healthy mice, accompanied by a reduction in SCFA concentrations. Furthermore, the intestinal tight junction barrier was disrupted, as indicated by increased intestinal paracellular permeability with an associated decrease in tight junction protein expressions. The authors opined that intestinal epithelial tight junction barrier dysfunction induced by antibiotics is associated with intestinal microbiota architecture alteration and an activated inflammasome. If these data do translate to humans, they have implications for the mental health impact of antibiotics. Changes to barrier function in humans would lead to a ‘leaky gut’. This, in turn, would allow molecules such as lipopolysaccharide to enter the bloodstream and initiate inflammation with increased activation of the HPA, rendering the individual vulnerable to depression. This is considered one of the mechanisms through which postinfective irritable bowel syndrome is associated with depression [111].

### Interaction with gut hormones

The gut peptides leptin and ghrelin have both been linked with mental health disorders [112, 113]. Leptin is predominantly made by adipose cells and enterocytes in the small intestine. Ghrelin, known as the hunger hormone, is produced in the stomach and recent studies indicate significant central effects, including regulation of anxiety levels. Alterations to the gut microbiota can result in changes to both leptin and ghrelin. Prebiotics, which promote the growth of good bacteria such as bifidobacteria, alter leptin and ghrelin levels, while antibiotics such as rifaxamin are also reported to change such peptides. No studies to date have explored in detail the link between antibiotic usage, gut peptides and the risk of mental health problems such as depression and anxiety. Such a study is essential to gain a greater mechanistic understanding of the linkage.

## Limitations

Most studies exploring the link between antibiotic use and mental health are based on a post hoc analysis where mental illness was not the primary outcome measure. The situation is further complicated because antibiotics with different structures are used to treat different infections, making comparisons across the antibiotic classes problematic. In all cases, the presence of infection is a potential confounding factor. Many infections are themselves associated with increased rates of depression, which is attributed to elevated cytokine levels. How antibiotics act to increase depression risk is based mainly on studies in rodents. There are few mechanistic studies conducted on humans.

## Ways forward

For novel antibiotics in clinical trials, a more detailed analysis of mental health adverse events should be collected. It is essential that such data be prospective rather than the current situation, where some adverse events simply emerge as case reports following the licencing of the drug. Furthermore, at a minimum, the impact of the novel antibiotic on the gut microbiota and the time frame for recovery should be monitored.

## Conclusions

Antibiotics, from a psychiatric perspective acting through the brain–gut–microbiota axis, can produce the good, the bad and the ugly. There is overwhelming evidence to support the view that antibacterial agents can have both a positive and a negative impact on mental health. The original antidepressants were derived from antimicrobial agents and remain effective antidepressants. However, with the increasing use of antibiotics, there are accumulating data indicating their negative impact on mental health. Overall, antibiotics increase the prevalence of both depression and anxiety. Rarely, they can also cause psychotic disorders with a schizophrenia‐like picture. Although most patients taking antibiotics do not suffer from an iatrogenic psychiatric disorder, some do, and as clinicians, we need to keep this in mind. The development of new antibiotics is primarily focused on antibiotic resistance. Still, efforts should be made to reduce off‐target effects resulting in mental health problems.

## Conflict of interest

The authors declare that there is no conflict of interest that could be perceived as prejudicing the impartiality of the research reported.
